# *N*-(5-Chloro-2-meth­oxy­phen­yl)benzene­sulfonamide

**DOI:** 10.1107/S1600536810041206

**Published:** 2010-10-20

**Authors:** Aziz-ur-Rehman, Shahzaman, Mehmet Akkurt, Muhammad Athar Abbasi, Islam Ullah Khan

**Affiliations:** a Department of Chemistry, Government College University, Lahore 54000, Pakistan; b Department of Physics, Faculty of Sciences, Erciyes University, 38039 Kayseri, Turkey

## Abstract

In the title compound, C_13_H_12_ClNO_3_S, the dihedral angle between the two aromatic rings is 73.94 (9)°. An intra­molecular C—H⋯O hydrogen bond occurs. In the crystal, inter­molecular N—H⋯O hydrogen bonds connect the mol­ecules to centrosymmetric dimers, forming an *R*_2_^2^(8) ring motif. The packing is consolidated by C—H⋯O hydrogen bonds and weak π–π inter­actions [centroid–centroid distances = 3.81 (3) and 3.81 (3) Å].

## Related literature

For the biological properties of sulfonamide derivatives, see: Berredjem *et al.* (2000 ▶); Lee & Lee (2002 ▶); Soledade *et al.* (2006 ▶); Xiao & Timberlake (2000 ▶). For related structures, see: Aziz-ur-Rehman *et al.* (2010*a* ▶,*b* ▶); Khan *et al.* (2010 ▶); Akkurt *et al.* (2010 ▶).
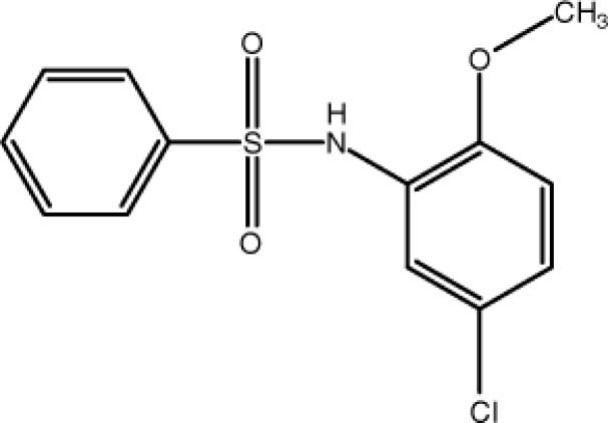


## Experimental

### 

#### Crystal data

C_13_H_12_ClNO_3_S
*M* *_r_* = 297.76
Triclinic, 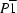

*a* = 8.2201 (2) Å
*b* = 8.9395 (2) Å
*c* = 10.5544 (2) Å
α = 77.206 (1)°
β = 76.366 (1)°
γ = 66.408 (1)°
*V* = 683.65 (3) Å^3^
*Z* = 2
Mo *K*α radiation
μ = 0.43 mm^−1^
*T* = 296 K
0.24 × 0.16 × 0.08 mm

#### Data collection

Bruker APEXII CCD diffractometer
12007 measured reflections
3333 independent reflections
2906 reflections with *I* > 2σ(*I*)
*R*_int_ = 0.023

#### Refinement

*R*[*F* ^2^ > 2σ(*F* ^2^)] = 0.035
*wR*(*F* ^2^) = 0.096
*S* = 1.02
3333 reflections
177 parameters
1 restraint
H atoms treated by a mixture of independent and constrained refinement
Δρ_max_ = 0.35 e Å^−3^
Δρ_min_ = −0.27 e Å^−3^

### 

Data collection: *APEX2* (Bruker, 2007 ▶); cell refinement: *SAINT* (Bruker, 2007 ▶); data reduction: *SAINT*; program(s) used to solve structure: *SHELXS97* (Sheldrick, 2008 ▶); program(s) used to refine structure: *SHELXL97* (Sheldrick, 2008 ▶); molecular graphics: *ORTEP-3 for Windows* (Farrugia, 1997 ▶); software used to prepare material for publication: *WinGX* (Farrugia, 1999 ▶) and *PLATON* (Spek, 2009 ▶).

## Supplementary Material

Crystal structure: contains datablocks global, I. DOI: 10.1107/S1600536810041206/bt5375sup1.cif

Structure factors: contains datablocks I. DOI: 10.1107/S1600536810041206/bt5375Isup2.hkl

Additional supplementary materials:  crystallographic information; 3D view; checkCIF report

## Figures and Tables

**Table 1:** Hydrogen-bond geometry (Å, °)

*D*—H⋯*A*	*D*—H	H⋯*A*	*D*⋯*A*	*D*—H⋯*A*
N1—H1⋯O1^i^	0.828 (16)	2.217 (16)	3.0096 (15)	160.2 (16)
C4—H4⋯O2^ii^	0.93	2.55	3.368 (3)	147
C8—H8⋯O2	0.93	2.34	2.9491 (17)	123
C13—H13*B*⋯O2^iii^	0.96	2.48	3.362 (3)	153

Symmetry codes: (i) 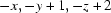; (ii) 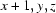; (iii) 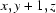.

**Fig. 1.**
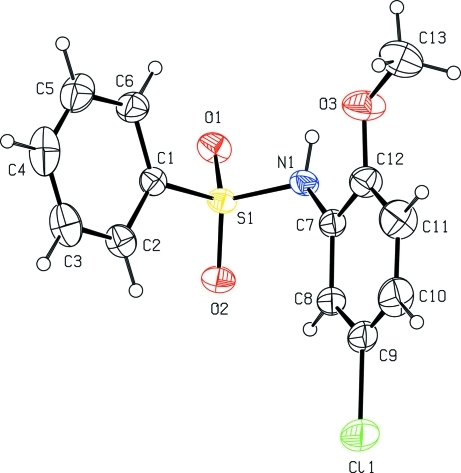
The title molecule drawn with displacement ellipsoids drawn at the 30% probability level.

**Fig. 2.**
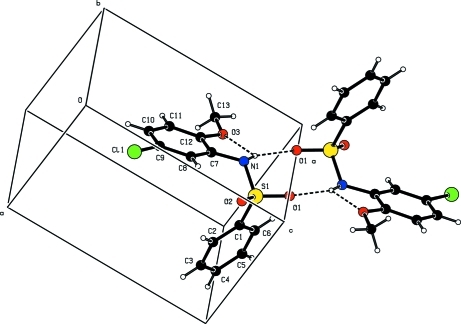
View of the dimeric N—H···O interactions between two moleculs in the unit cell.

## supplementary crystallographic information

### Comment

Sulfonamide is found in a number of synthetic as well as natural compounds. These molecules posses many biological activities *e.g.*, herbicidal, anti-malarial, anti-convulsant and anti- hypertensive (Soledade *et al.*, 2006; Xiao & Timberlake, 2000; Berredjem *et al.*, 2000; Lee & Lee, 2002). In the present paper, the structure of *N*-(5-chloro-2-methoxyphenyl) benzenesulfonamide has been determined as part of a research program involving the synthesis and biological evaluation of sulfur containing compounds.

In the title molecule (I), (Fig. 1), both sulfonamido-O atoms lie on the opposite side of the S-bound phenyl ring to the sulfonamido-N1 atom [the O1–S1–C1–C6, O2–S1–C1–C2 and N1–S1–C1–C2 torsion angles are -34.32 (13), 14.43 (13) and -101.83 (12) °, respectively]. The dihedral angle formed between the phenyl (C1–C6) and benzene (C7–C12) rings in (I) is 73.94 (9) °.

The molecules of (I) are dimerized due to the intermolecular N—H···O hydrogen bonding (Table 1, Fig. 2) producing a *R*_2_^2^(8) ring motif.

In addition, there are C—H···O hydrogen bonds, as well as π-π interactions [*Cg*1···*Cg*1(1 - *x*, -*y*, 2 - *z*) = 3.8163 (11) Å and cg2···*Cg*2(-*x*, 1 - *y*, 1 - *z*) = 3.9472 (12) Å; where *Cg*1 and *Cg*2 are centroids of the phenyl and benzene rings (C1–C6 and C7–C12), respectively], between the aromatic rings of each dimer.

### Experimental

A mixture benzenesulfonyl chloride (10.0 mmol; 1.45 ml), 5-chloro-2-methoxy aniline (10.0 mmol; 1.47 g), aqueous sodium carbonate (10%; 20.0 ml) and water (25 ml) was stirred for one and half hour at room temperature. The crude mixture was washed with water and dried. The product was dissolved in methanol and recrystallized by slow evaporation of the solvent, to generate colourless crystal of *N*-(5-chloro-2-methoxyphenyl)benzenesulfonamide in 71% yield.

### Refinement

The amino H atom is located in a difference Fourier map and refined freely. The remaining H atoms were positioned geometrically (C—H = 0.93 and 0.96 Å) and allowed to ride on their parent atoms, with *U*_iso_(H) = 1.2*U*_eq_(C).

### Crystal data

**Table:**

C_13_H_12_ClNO_3_S	*Z* = 2
*M**_r_* = 297.76	*F*(000) = 308
Triclinic, *P*1	*D*_x_ = 1.446 Mg m^−^^3^
Hall symbol: -P 1	Mo *K*α radiation, λ = 0.71073 Å
*a* = 8.2201 (2) Å	Cell parameters from 6946 reflections
*b* = 8.9395 (2) Å	θ = 2.5–28.2°
*c* = 10.5544 (2) Å	µ = 0.43 mm^−^^1^
α = 77.206 (1)°	*T* = 296 K
β = 76.366 (1)°	Prism, colourless
γ = 66.408 (1)°	0.24 × 0.16 × 0.08 mm
*V* = 683.65 (3) Å^3^	

### Data collection

**Table:**

Bruker APEXII CCD diffractometer	2906 reflections with *I* > 2σ(*I*)
Radiation source: sealed tube	*R*_int_ = 0.023
graphite	θ_max_ = 28.3°, θ_min_ = 4.0°
φ and ω scans	*h* = −10→10
12007 measured reflections	*k* = −11→11
3333 independent reflections	*l* = −14→12

### Refinement

**Table:**

Refinement on *F*^2^	Primary atom site location: structure-invariant direct methods
Least-squares matrix: full	Secondary atom site location: difference Fourier map
*R*[*F*^2^ > 2σ(*F*^2^)] = 0.035	Hydrogen site location: inferred from neighbouring sites
*wR*(*F*^2^) = 0.096	H atoms treated by a mixture of independent and constrained refinement
*S* = 1.01	*w* = 1/[σ^2^(*F*_o_^2^) + (0.0504*P*)^2^ + 0.1401*P*] where *P* = (*F*_o_^2^ + 2*F*_c_^2^)/3
3333 reflections	(Δ/σ)_max_ = 0.001
177 parameters	Δρ_max_ = 0.35 e Å^−^^3^
1 restraint	Δρ_min_ = −0.27 e Å^−^^3^

### Special details

**Table:**

Geometry. Bond distances, angles *etc*. have been calculated using the rounded fractional coordinates. All su's are estimated from the variances of the (full) variance-covariance matrix. The cell e.s.d.'s are taken into account in the estimation of distances, angles and torsion angles
Refinement. Refinement on *F*^2^ for ALL reflections except those flagged by the user for potential systematic errors. Weighted *R*-factors *wR* and all goodnesses of fit *S* are based on *F*^2^, conventional *R*-factors *R* are based on *F*, with *F* set to zero for negative *F*^2^. The observed criterion of *F*^2^ > σ(*F*^2^) is used only for calculating -*R*-factor-obs *etc*. and is not relevant to the choice of reflections for refinement. *R*-factors based on *F*^2^ are statistically about twice as large as those based on *F*, and *R*-factors based on ALL data will be even larger.

### Fractional atomic coordinates and isotropic or equivalent isotropic displacement parameters (Å^2^)

**Table:**

	*x*	*y*	*z*	*U*_iso_*/*U*_eq_	
Cl1	0.29733 (8)	0.24190 (8)	0.34117 (5)	0.0934 (2)	
S1	0.14406 (4)	0.27960 (4)	0.88801 (3)	0.0380 (1)	
O1	0.03295 (14)	0.31471 (13)	1.01239 (9)	0.0510 (3)	
O2	0.13974 (14)	0.15335 (13)	0.82861 (10)	0.0506 (3)	
O3	0.1709 (2)	0.71184 (16)	0.69335 (12)	0.0734 (5)	
N1	0.08081 (16)	0.45288 (14)	0.78854 (10)	0.0429 (3)	
C1	0.36916 (18)	0.23297 (16)	0.90100 (12)	0.0398 (4)	
C2	0.5039 (2)	0.11582 (19)	0.83059 (15)	0.0521 (5)	
C3	0.6805 (2)	0.0782 (2)	0.8436 (2)	0.0696 (6)	
C4	0.7178 (3)	0.1563 (3)	0.9253 (2)	0.0767 (7)	
C5	0.5824 (3)	0.2731 (3)	0.9938 (2)	0.0731 (7)	
C6	0.4049 (2)	0.3136 (2)	0.98279 (16)	0.0557 (5)	
C7	0.16189 (18)	0.47011 (17)	0.65422 (12)	0.0422 (4)	
C8	0.1896 (2)	0.35789 (19)	0.57270 (13)	0.0505 (4)	
C9	0.2635 (2)	0.3861 (2)	0.44138 (15)	0.0616 (5)	
C10	0.3044 (3)	0.5227 (3)	0.39090 (16)	0.0756 (7)	
C11	0.2737 (3)	0.6360 (3)	0.47153 (17)	0.0739 (7)	
C12	0.2046 (2)	0.6098 (2)	0.60401 (14)	0.0541 (5)	
C13	0.1961 (3)	0.8625 (3)	0.6504 (3)	0.0875 (9)	
H1	0.058 (2)	0.5318 (19)	0.8273 (16)	0.052 (4)*	
H2	0.47690	0.06350	0.77580	0.0630*	
H3	0.77380	0.00000	0.79700	0.0830*	
H4	0.83650	0.12960	0.93410	0.0920*	
H5	0.61010	0.32540	1.04810	0.0880*	
H6	0.31220	0.39270	1.02900	0.0670*	
H8	0.15940	0.26520	0.60520	0.0600*	
H10	0.35290	0.53940	0.30240	0.0910*	
H11	0.29940	0.73070	0.43700	0.0890*	
H13A	0.32050	0.84190	0.61440	0.1310*	
H13B	0.16150	0.92250	0.72360	0.1310*	
H13C	0.12340	0.92620	0.58390	0.1310*	

### Atomic displacement parameters (Å^2^)

**Table:**

	*U*^11^	*U*^22^	*U*^33^	*U*^12^	*U*^13^	*U*^23^
Cl1	0.1058 (4)	0.0954 (4)	0.0594 (3)	0.0032 (3)	−0.0179 (2)	−0.0448 (3)
S1	0.0403 (2)	0.0368 (2)	0.0370 (2)	−0.0163 (1)	0.0011 (1)	−0.0095 (1)
O1	0.0538 (6)	0.0510 (6)	0.0416 (5)	−0.0206 (5)	0.0098 (4)	−0.0100 (4)
O2	0.0566 (6)	0.0459 (5)	0.0577 (6)	−0.0253 (5)	−0.0063 (5)	−0.0146 (4)
O3	0.1093 (11)	0.0642 (8)	0.0596 (7)	−0.0505 (8)	0.0026 (7)	−0.0160 (6)
N1	0.0491 (6)	0.0385 (6)	0.0360 (5)	−0.0107 (5)	−0.0019 (4)	−0.0111 (4)
C1	0.0435 (7)	0.0381 (6)	0.0381 (6)	−0.0193 (5)	−0.0049 (5)	0.0003 (5)
C2	0.0465 (8)	0.0504 (8)	0.0515 (8)	−0.0145 (6)	−0.0008 (6)	−0.0058 (6)
C3	0.0437 (8)	0.0664 (11)	0.0793 (11)	−0.0144 (8)	0.0003 (8)	0.0052 (9)
C4	0.0524 (10)	0.0787 (13)	0.0975 (14)	−0.0378 (10)	−0.0255 (10)	0.0310 (11)
C5	0.0829 (13)	0.0731 (12)	0.0857 (13)	−0.0494 (11)	−0.0375 (11)	0.0098 (10)
C6	0.0643 (9)	0.0529 (8)	0.0591 (9)	−0.0286 (7)	−0.0153 (7)	−0.0068 (7)
C7	0.0419 (7)	0.0437 (7)	0.0351 (6)	−0.0092 (5)	−0.0058 (5)	−0.0073 (5)
C8	0.0535 (8)	0.0491 (8)	0.0420 (7)	−0.0073 (6)	−0.0100 (6)	−0.0131 (6)
C9	0.0611 (9)	0.0675 (10)	0.0397 (7)	0.0007 (8)	−0.0109 (6)	−0.0189 (7)
C10	0.0797 (12)	0.0875 (14)	0.0370 (7)	−0.0161 (10)	0.0030 (7)	−0.0062 (8)
C11	0.0871 (13)	0.0748 (12)	0.0503 (9)	−0.0341 (10)	0.0042 (8)	0.0014 (8)
C12	0.0602 (9)	0.0554 (9)	0.0442 (7)	−0.0219 (7)	−0.0022 (6)	−0.0080 (6)
C13	0.1028 (17)	0.0654 (12)	0.1064 (17)	−0.0453 (12)	−0.0112 (13)	−0.0149 (11)

### Geometric parameters (Å, °)

**Table:**

Cl1—C9	1.7424 (17)	C7—C8	1.380 (2)
S1—O1	1.4296 (10)	C8—C9	1.387 (2)
S1—O2	1.4227 (12)	C9—C10	1.358 (3)
S1—N1	1.6323 (12)	C10—C11	1.375 (3)
S1—C1	1.7588 (16)	C11—C12	1.386 (2)
O3—C12	1.361 (2)	C2—H2	0.9300
O3—C13	1.404 (3)	C3—H3	0.9300
N1—C7	1.4205 (16)	C4—H4	0.9300
N1—H1	0.828 (16)	C5—H5	0.9300
C1—C2	1.380 (2)	C6—H6	0.9300
C1—C6	1.384 (2)	C8—H8	0.9300
C2—C3	1.386 (3)	C10—H10	0.9300
C3—C4	1.374 (3)	C11—H11	0.9300
C4—C5	1.370 (3)	C13—H13A	0.9600
C5—C6	1.383 (3)	C13—H13B	0.9600
C7—C12	1.391 (2)	C13—H13C	0.9600
			
Cl1···C6^i^	3.6473 (17)	C7···Cl1^iii^	3.6152 (17)
Cl1···C2^ii^	3.6292 (17)	C7···C9^iii^	3.520 (2)
Cl1···C7^iii^	3.6152 (17)	C8···C8^iii^	3.566 (2)
Cl1···H2^ii^	2.9600	C8···O2	2.9491 (17)
Cl1···H13A^iv^	3.0500	C8···C9^iii^	3.524 (2)
S1···H8	2.9900	C9···C8^iii^	3.524 (2)
O1···N1^v^	3.0096 (15)	C9···C7^iii^	3.520 (2)
O1···O1^v^	3.0957 (15)	C13···O2^xi^	3.362 (3)
O1···O3^v^	3.1699 (16)	C3···H11^iv^	3.0800
O2···C13^vi^	3.362 (3)	C11···H13A	2.8100
O2···C4^vii^	3.368 (3)	C11···H13C	2.7900
O2···C8	2.9491 (17)	C13···H11	2.5800
O2···C4^viii^	3.382 (2)	H1···O3	2.241 (17)
O3···O1^v^	3.1699 (16)	H1···O1^v^	2.217 (16)
O3···N1	2.6284 (19)	H2···O2	2.5100
O1···H6	2.7000	H2···Cl1^ii^	2.9600
O1···H1^v^	2.217 (16)	H4···O2^ix^	2.5500
O2···H13B^vi^	2.4800	H6···O1	2.7000
O2···H4^vii^	2.5500	H8···S1	2.9900
O2···H8	2.3400	H8···O2	2.3400
O2···H2	2.5100	H11···C13	2.5800
O3···H1	2.241 (17)	H11···H13A	2.3700
N1···O3	2.6284 (19)	H11···H13C	2.3800
N1···O1^v^	3.0096 (15)	H11···C3^iv^	3.0800
C1···C3^viii^	3.499 (2)	H13A···C11	2.8100
C2···Cl1^ii^	3.6292 (17)	H13A···H11	2.3700
C3···C1^viii^	3.499 (2)	H13A···Cl1^iv^	3.0500
C4···O2^ix^	3.368 (3)	H13B···O2^xi^	2.4800
C4···O2^viii^	3.382 (2)	H13C···C11	2.7900
C6···Cl1^x^	3.6473 (17)	H13C···H11	2.3800
			
O1—S1—O2	118.95 (7)	C10—C11—C12	120.3 (2)
O1—S1—N1	105.06 (6)	C7—C12—C11	119.51 (16)
O1—S1—C1	109.15 (7)	O3—C12—C7	114.94 (13)
O2—S1—N1	108.21 (6)	O3—C12—C11	125.55 (17)
O2—S1—C1	107.73 (7)	C1—C2—H2	121.00
N1—S1—C1	107.19 (7)	C3—C2—H2	121.00
C12—O3—C13	119.30 (17)	C2—C3—H3	120.00
S1—N1—C7	122.57 (10)	C4—C3—H3	120.00
C7—N1—H1	115.2 (11)	C3—C4—H4	120.00
S1—N1—H1	110.4 (11)	C5—C4—H4	120.00
S1—C1—C6	118.85 (11)	C4—C5—H5	120.00
S1—C1—C2	118.88 (12)	C6—C5—H5	120.00
C2—C1—C6	122.27 (16)	C1—C6—H6	121.00
C1—C2—C3	118.25 (15)	C5—C6—H6	121.00
C2—C3—C4	120.09 (18)	C7—C8—H8	121.00
C3—C4—C5	120.9 (2)	C9—C8—H8	121.00
C4—C5—C6	120.5 (2)	C9—C10—H10	120.00
C1—C6—C5	118.07 (17)	C11—C10—H10	120.00
N1—C7—C12	117.82 (12)	C10—C11—H11	120.00
N1—C7—C8	121.96 (13)	C12—C11—H11	120.00
C8—C7—C12	120.14 (12)	O3—C13—H13A	109.00
C7—C8—C9	118.69 (15)	O3—C13—H13B	109.00
Cl1—C9—C8	117.57 (13)	O3—C13—H13C	109.00
C8—C9—C10	121.73 (16)	H13A—C13—H13B	109.00
Cl1—C9—C10	120.68 (13)	H13A—C13—H13C	109.00
C9—C10—C11	119.57 (17)	H13B—C13—H13C	109.00
			
O1—S1—N1—C7	179.00 (12)	C1—C2—C3—C4	0.2 (3)
O2—S1—N1—C7	−52.96 (14)	C2—C3—C4—C5	−0.7 (3)
C1—S1—N1—C7	62.98 (14)	C3—C4—C5—C6	0.6 (3)
O1—S1—C1—C2	144.89 (11)	C4—C5—C6—C1	0.0 (3)
O2—S1—C1—C2	14.43 (13)	C12—C7—C8—C9	1.2 (2)
N1—S1—C1—C2	−101.83 (12)	N1—C7—C12—O3	3.1 (2)
O1—S1—C1—C6	−34.32 (13)	C8—C7—C12—O3	179.75 (16)
O2—S1—C1—C6	−164.78 (11)	C8—C7—C12—C11	0.5 (3)
N1—S1—C1—C6	78.96 (12)	N1—C7—C12—C11	−176.09 (18)
C13—O3—C12—C7	−175.04 (19)	N1—C7—C8—C9	177.65 (15)
C13—O3—C12—C11	4.1 (3)	C7—C8—C9—C10	−1.8 (3)
S1—N1—C7—C12	−135.26 (14)	C7—C8—C9—Cl1	179.83 (13)
S1—N1—C7—C8	48.2 (2)	C8—C9—C10—C11	0.6 (3)
C2—C1—C6—C5	−0.4 (2)	Cl1—C9—C10—C11	178.93 (19)
C6—C1—C2—C3	0.3 (2)	C9—C10—C11—C12	1.2 (4)
S1—C1—C2—C3	−178.87 (12)	C10—C11—C12—C7	−1.7 (3)
S1—C1—C6—C5	178.76 (14)	C10—C11—C12—O3	179.1 (2)

Symmetry codes: (i) *x*, *y*, *z*−1; (ii) −*x*+1, −*y*, −*z*+1; (iii) −*x*, −*y*+1, −*z*+1; (iv) −*x*+1, −*y*+1, −*z*+1; (v) −*x*, −*y*+1, −*z*+2; (vi) *x*, *y*−1, *z*; (vii) *x*−1, *y*, *z*; (viii) −*x*+1, −*y*, −*z*+2; (ix) *x*+1, *y*, *z*; (x) *x*, *y*, *z*+1; (xi) *x*, *y*+1, *z*.

### Hydrogen-bond geometry (Å, °)

**Table:**

*D*—H···*A*	*D*—H	H···*A*	*D*···*A*	*D*—H···*A*
N1—H1···O1^v^	0.828 (16)	2.217 (16)	3.0096 (15)	160.2 (16)
C4—H4···O2^ix^	0.93	2.55	3.368 (3)	147
C8—H8···O2	0.93	2.34	2.9491 (17)	123
C13—H13B···O2^xi^	0.96	2.48	3.362 (3)	153

Symmetry codes: (v) −*x*, −*y*+1, −*z*+2; (ix) *x*+1, *y*, *z*; (xi) *x*, *y*+1, *z*.
